# Utilization of kinase inhibitors as novel therapeutic drug targets: A review

**DOI:** 10.32604/or.2022.027549

**Published:** 2023-02-03

**Authors:** SUCHITRA NISHAL, VIKAS JHAWAT, SUMEET GUPTA, PARMITA PHAUGAT

**Affiliations:** 1 Department of Pharmacy, School of Medical and Allied Sciences, GD Goenka University, Gurugram, India; 2 Department of Pharmaceutical Sciences, Maharishi Markandeshwar (Deemed to be) University, Mullana, Ambala, Haryana, India

**Keywords:** Kinase inhibitors, Targeted, Drug designing, Anti-cancer, Auto-immune diseases

## Abstract

Kinase inhibitors are a significant and continuously developing division of target therapeutics. The drug discovery and improvement efforts have examined numerous attempts to target the signaling pathway of kinases. The Kinase inhibitors have been heralded as a game-changer in cancer treatment. For developing kinase inhibitors as a treatment for various non-malignant disorders like auto-immune diseases, is currently undergoing extensive research. It may be beneficial to investigate whether cell-specific kinase inhibitor administration enhances therapeutic efficacy and decreases adverse effects. The goal of the current review is to gain insight into the role of kinase inhibitors in facilitating effective target drug delivery for the treatment of various anti-inflammatory, auto-immune, and anticancer disorders. The aim of this review is also to shed light on drug discovery approaches for kinase inhibitors, their mode of action, and delivery approaches. The variation in the binding of kinases bestows different target approaches in drug design, which can be employed for designing the targeted molecules. Several target sites have been studied, exceeding the design of drugs for various diseases like cancer, Alzheimer’s, rheumatoid arthritis, etc. Diverse delivery approaches have also been studied for the targeted application of kinase inhibitors.

## Introduction

Cellular metabolism, cell cycle regulation, cellular endurance, and differentiation are the essential functions conducted by kinases [1–3]. Nowadays, considerable attention has been given to research on kinases. The signaling pathways mediated by protein kinases have been related to a variety of diseases, such as diabetes, inflammation, and cancer [4]. The kinases are categorized into a wide group lipid kinases, carbohydrates, and protein kinases. According to human genome sequencing, approximately 2% of the human genome codes for protein kinases, which are further classified as families and subfamilies. The major kinases found in mammalian signaling pathways are lipid kinases, tyrosine kinases, serine/threonine kinases, and dual kinases (Ser/Thr and Ty). Protein kinases are enzymes that use the end γ-phosphate group from ATP to phosphorylate serine, tyrosine, or threonine residues in other proteins. Phosphorylation changes target protein activity by controlling signaling pathways by amplification, cellular location, or interaction with other controlling proteins [5]. The study on human kinases revealed that there are 518 protein kinases grouped into families reliant on their biological functions as well as statistical sequence evaluation [6].

Kinase inhibitors are small molecules that inhibit kinases and are of much use in therapeutic and diagnostic fields. According to recent research, Kinase inhibitors serve as a potential target for treating various diseases like autoimmune disorders, cardiovascular diseases, cancer, and inflammatory disorders [2]. As of November 24, 2022, the US FDA had approved 72 small molecules that are therapeutic protein kinase inhibitors. All drugs that have been approved by the FDA work best when taken orally, with the exception of temsirolimus, trilaciclib, and netarsudil [7,8] (https://brimr.org/protein-kinase-inhibitors/). Novel biologic agents, such as monoclonal antibodies, can also be employed for targeting specific kinases. However, target non-specificity and toxicity are the principal disadvantages associated with kinase inhibitors. Small molecules from natural sources may act together with proteins and function as signaling molecules, which can be further employed for human health. The kinase inhibitors from various plant products help treat several disorders (Table 1).

**TABLE 1:** Describes the kinase inhibitors obtained from various plant sources

S.No.	Natural plant category		Examples	Primary target	References
1.	Polyphenols	Flavonoid analogues	Quercetin, Apigenin	CK2 inhibitor, Ser/Thr protein kinase	[9,10]
		Anthraquinones	Rhein, Emodin	IKKβ inhibitor; tyrosine kinase p56 inhibitor	[11,12]
			Quinone naphthazarin	Aurora kinase A & B	[13]
		Phenolic acids	Tannic acid, Ellagic acid	Protein kinase C, CK2	[14,15]
		Lignans	Arctigenein, Honokilol	ERK1/2, p38, EGFR inhibitor	[16,17]
		Coumarins	Daphnetin, Coumesterol	EGFR inhibitor; CK2 inhibitor	[18,19]
		Other polyphenols	Resveratrol	p38, PKB, PKC, Src, ERK1/2, JNK1/2, PI3K, and IKK	[20]
2.	Indolocarbazole analogues		Staurosporine	PKA, PKC, PKG, GSK-3β, MLCK, CAMKII, and CDK2,	[21]
3.	Furanosteroid analogues		Halenaquinone	pp60^V-SRC^	[22]
4.	Purine analogues		Lymphostein	Lymphocyte-specific protein tyrosine kinase	[23]
5.	Alkaloids		Sanguinarine	BCL-2, MAPKs, Akt, NF-κB, ROS, and microRNAs	[24,25]
			Steroidal alkaloid tomatidine	Type I interferon-stimulated genes	[26]
6.	Other natural substances		Hypothemycin; Harmine	PDGFRα & β , SRC, ERK1/2, PKD1, TRKA, & TRKB; MOA	[27,28]

In an *in vivo* study performed on cancer cell line 85As2 (Gastric cancer cell line), the steroidal alkaloid tomatidine as well as tomatidine rich tomato leaf extract exhibited reduction in proliferation of 85As2 cells as a result of dysregulation of type 1 interferon-stimulated genes [26]. As a result, several marine natural chemicals have been exercised as lead drug composites that progress in future medicines. Around 22 kinase inhibitors are synthesized from marine bacteria, for instance, Bacillus and Streptomyces species; 16 from marine fungi sources like Penicillium, Aspergillus, etc.; two new kinase inhibitors from deep-sea soft corals like *Cladiella australis*, *Cladiella pachyclados*; 14 kinase inhibitors from marine animals like sea cucumber, marine echinoderm, etc.; 10 from marine algae-like cyanobacteria; 42 from marine sponges [5].

### Role of kinase inhibitors in drug design

**FIGURE 1:**
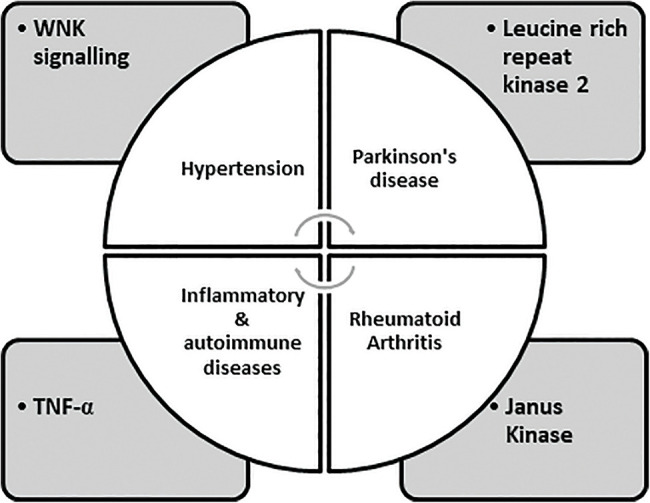
Expressing targets employed in the development of kinase inhibitors [7].

Kinase inhibitors are a rapidly growing and important category of target therapeutics. To target kinases signaling, drug discovery and development initiatives have looked at a range of approaches. The role of kinases in drug designing and as delivery systems has been described as follows:

Many protein kinases are part of “cascade systems,” where multiple protein kinases sequentially activate each other. A kinase cascade plays a vital role in amplification, diversification of signal effects, and permission for grouping signaling pathways to form networks. Because kinase cascades exist, it is possible to find and produce drugs that are not kinase inhibitors but prevent kinases from being triggered due to the subsequent “upstream” kinase in the cascade through attachment to them. PD98059 is certainly the first kinase inhibitor developed for the Ras-Raf MEK-ERK cascade, which acts through binding to MEK1 and then blocking its stimulation via Raf [29,30]. The dual-specificity protein kinases (MEK1/2) that block the RAS-Raf-MEK MAP kinase pathway are trametinib, binimetinib, cobimetinib, and selumetinib. Among these, binimetinib and cobimetinib are used in combination with encorafenib and vemurafenib, respectively for curing melanoma. Selumetinib is employed for Von Recklinghausen disease [7]. Raf, Src, epidermal growth factor, and breakpoint cluster area are all kinase targets. Early on in the investigation of oncogenic proteins, Abelson’s kinase (bcr-Abl) was discovered. Clinical usage of these inhibitors has also resulted in the growth of drug-resistant malignancies as additional consequences [31,32]. The detection alongside subsequent exploitation of numerous configurational positions of kinases is part of the tale of developing these kinase inhibitors. Previously, selectivity had been identified as a problem during the development of kinase inhibitors that bind at the ATP (Adenosine Triphosphate) pocket. As ATP is a cofactor required for the functioning of the kinase, developmental pressure has been applied to keep the binding site of discrete kinases in a general shape and chemical similarity. As a result, the active sites avoiding the direct binding of ATP or kinase conformations with more structural and chemical heterogeneity have been employed. The activation and inactivation modes of kinases allow for the utilization of conformational variability. A rationale based on the structure was also proposed to prevent the formation of drug-resilient mutations. Since the inactive version of a kinase does not require binding with the conjoint substrate ATP, it has been found to have higher structural diversity. A pioneer kinase inhibitor, Imatinib, exhibits binding to the inactive forms of PDGF, c-Kit, and Abl kinases [33–36]. Imatinib is used to treat chronic myelogenous leukemia as it causes inhibition of BCR-Abl, an oncogenic tyrosine kinase fusion protein also employed to manage gastrointestinal tumors and myeloproliferative disorders caused by inhibition of c-Kit tyrosine kinase and PDGF receptors, respectively. As a result, oncology happens to be the therapeutic area of choice for the vast majority of drug development efforts for kinase inhibitors [33,37]. Most of the small protein kinase inhibitors that have been approved or are on the verge of getting approval for their clinical use, along with the majority of kinase inhibitors currently in the clinical trial phase, intend to target tyrosine kinases and are employed for the treatment of varied cancers. The global market for kinase inhibitors is increasing gradually. Although cancer will likely remain a top priority for kinase medication development for several years, the number of kinase inhibitors in clinical studies for many other disorders has expanded [38–40]. For example, Janus Kinase inhibitors such as tofacitinib and ruxolitinib have been approved to treat rheumatoid arthritis and myelofibrosis, respectively [29,41]. Inhibition of anaplastic lymphoma kinase (ALK) by six approved molecules, i.e., Alectinib, Brigatinib, Ceritinib, Crizotinib, Entrectinib, and Lorlatinib, has been employed for the treatment of NSCLC (Non-Small Cell Lung Cancer) (**link**). Targets used for developing kinase inhibitors for diseases other than cancer are described in Fig. 1.

WNKs direct the phosphorylation and activation of two interrelated protein kinases: SPAK (STE20/SPS1-related Proline/Alanine-Rich Kinase), OSRK1 (Oxidative Stress Responsive Kinase1), leading to the discovery that they control blood pressure. The unique location of a catalytic lysine residue on WNK isoforms might be of potential use in contrast to many other kinases to build WNK-specific ATP-competitive inhibitors, opening up a new avenue for developing better blood pressure medications. Although it is uncertain which WNK isoform of the four mammalian isoforms is possibly essential to be blocked to lower NCC (Na+/Cl− co-transporter) and NKCC2 (Na+/K+/2Cl^−^ co-transporter-2) activity in the kidney. Additionally, in some studies, WNK4 was a negative controller of WNK1 signaling; signifying that blockage of this isoform might enhance renal salt reassimilation and blood pressure [42]. Patients with mutations of LRKK2 acquire signs of disease that are identical to both the onset and progression of idiopathic Parkinson’s syndrome. LRKK2 is a multidomain protein with a large GTPase and a kinase domain of 2527 residues. Numerous particular LRKK2 inhibitors have been developed for the treatment of Parkinson’s syndrome. Biological drugs, like adalimumab tend to neutralize TNF-α and have a specific role in treating many inflammatory disorders like Crohn’s disease, psoriatic arthritis, and rheumatoid arthritis. Several Ser/Thr specific protein kinases are involved in the Myeloid cell signaling pathway, which regulates translation, transcription, processing, and secretion of TNF-α along with other pro-inflammatory cytokines. Many serine/threonine-specific protein kinases are involved in these systems, which regulate TNF and other proinflammatory cytokines’ transcription, translation, processing, and secretion. However, it is challenging to develop advanced drugs to target these signaling pathways owing to the complexity in deciding the target protein kinase due to the production of cytokines other than IL-6/12 and TNF-α [29,43,44].

## Targets for Developing Kinase Inhibitors

WNKs direct the phosphorylation and activation of two interrelated protein kinases: SPAK (STE20/SPS1-related Proline/Alanine-Rich Kinase), OSRK1 (Oxidative Stress Responsive Kinase1), leading to the discovery that they control the blood pressure. The ability to build WNK-specific ATP-competitive inhibitors using the unique location of a catalytic lysine residue on WNK isoforms, as opposed to many other kinases, opens up a new avenue for developing better blood pressure medications. Though, it is uncertain which WNK isoform of the four mammalian isoforms is possibly essential to be blocked to lower NCC (Na+/Cl− co-transporter) and NKCC2 (Na+/K+/2Cl− co-transporter-2) activity in the kidney. Additionally, in some studies, WNK4 was a negative controller of WNK1 signaling, signifying that blockage of this isoform might enhance the renal salt re-assimilation and blood pressure [42]. Human inherited blood pressure diseases are caused by mutations in WNK1, WNK4, NCC, and NKCC2, emphasizing their significance. According to recent studies, SPAK and OSR1 are interesting therapeutic candidates for the treatment of hypertension since blocking these enzymes will decrease NCC and NKCC2 activity, and hence prevent renal salt reabsorption [45–47]. In a variety of cancers, aurora kinases are overexpressed. When Aurora-A is overexpressed in upper gastrointestinal adenocarcinomas *in vitro*, camptothecin cannot cause apoptosis, increasing the lifespan of cancerous cells [48]. Members of serine/threonine kinase family, aurora kinase-A/B/C are crucial mitotic regulators necessary for genomic stability [49]. The discovery of protein kinase drugs may be traced back to the discovery of Src, the first oncogenic type protein kinase, nearly 25 years ago. It directed the global research attempts to explore the genomic and proteomic associations of protein kinases in various diseases, the mechanism of signal transduction through protein kinases, approaches for stimulation and inhibition of protein kinases, and the discovery of chemical entities based on new protein kinase inhibitors. According to recent human genome sequencing, protein kinases are currently recognized as a superclass of therapeutic targets with over 500 members. Table 2 can describe the classes of protein kinases [29,45].

**TABLE 2:** Protein kinase classes and examples

Protein kinases			
Receptor tyrosine kinase	Non-receptor tyrosine kinase	Receptor serine/Threonine kinase	Non-receptor serine/Threonine kinase
Epidermal growth factor receptor (EGFR) Fibroblast growth factor receptor (FGFR) Vascular endothelial growth factor receptor (VEGFR) Platelet-derived growth factor receptor (PDGFR) Colony-stimulating growth factor receptor (CSGFR) Nerve growth factor receptor (NGFR) Insulin-like growth factor receptor (ILGFR) Insulin receptor	Src/Src family Abl & BCR-Abl C-terminal Src kinase (CSK) Janus Kinase [50]	Transforming growth factor beta receptors (TGF-β) [51,52]	cAMP-dependent protein kinase Cyclin-dependent kinase Phosphoinositol-3-kinase Protein Kinase C Mitogen-activated protein kinase Rho-dependent protein kinase Akt mTOR (mammalian target of rapamycin)

**FIGURE 2:**
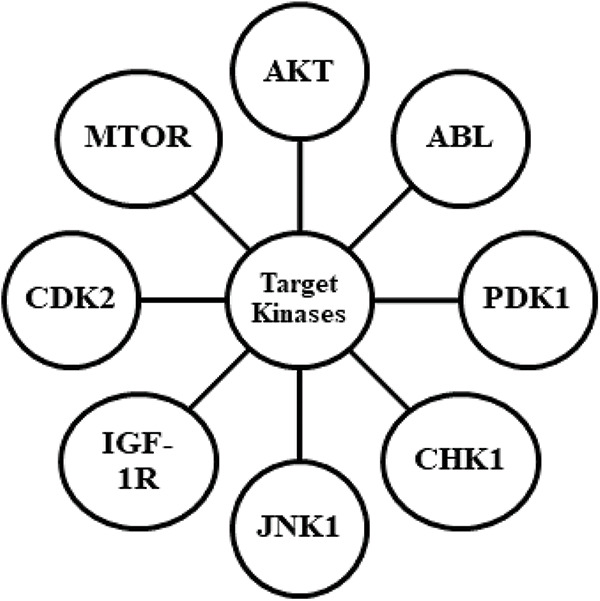
Describing the targets for kinase inhibitors for designing drug candidates.

Given the vast number of druggable kinase foci and the solid structural resemblance between the ATP binding sites of diverse kinase targets, there is increasing interest in studying inhibitors with various molecular roles to find higher selectivity profiles. A large proportion of small molecule kinase inhibitor (SMKIs) approved so far are ATP competitive inhibitors. The Type I inhibitors bind to the pivot area of the active kinase, i.e., DFG motif, and compete with ATP inhibitors in every respect. Type II inhibitors present binding to an enzymatically dormant kinase, i.e., DFG-out, and provide stabilization. Type III inhibitors retain the kinase in the enzymatically inactive DFG-out confirmation by occupying the neighbouring inducible pocket or rear pocket. Type III inhibitors regulate kinase activity in an allosteric manner by attaching distantly from the catalytic ATP-binding site. These can be further divided into two subtypes, namely Type IIIA and Type IIIB. MEK1/2 inhibitors, which bind at a particular cavity close to the ATP-binding site, are some of the well-studied type III inhibitors. Trametinib, Selumetinib, Binimetinib, and Cobimetinib are allosteric inhibitors of MEK and are among the MEK inhibitors currently approved by the FDA [38,53–56]. The regions outside the ATP-binding sites are the focus of Type IV inhibitors, also known as substrate-directed inhibitors, which are allosteric inhibitors that do not overlap with Type III inhibitors. Sirolimus, temsirolimus, and everolimus are examples of Type IV inhibitors with FDA approval. Type V is bivalent inhibitors that stably bind to active kinase sites and peptide motifs that correspond to the substrate that the kinase is targeting. Bivalent kinase inhibitors are typically very effective and very selective because they specifically target the cSrc tyrosine kinase, and MAP kinases have been found in several investigations. Since it has not yet received approval for use in clinical trials, this kind of inhibitor is still being researched. Because of their ability to interact with the cysteine nucleophiles in the pivot area of the ATP-pockets and form a covalent adduct, afatinib, neratinib, ibrutinib, and dacomitinib are covalent inhibitors, and are categorized under Type VI. The Type VII inhibitors are classified as non-classical allosteric inhibitors that specifically target a receptor tyrosine kinase’s extracellular domain. These are less potent than other types of inhibitors and do not directly prevent binding at the kinase domain/ligand-polypeptide binding site. SSR128129E and WRG-28 are two examples of this type of kinase inhibitor, which inhibits the FGFR (extracellular domain of fibroblast growth factor receptor) family and DDRs (discoidin domain receptors), respectively [7,41,53,56,57]. The structured kinase mapping analyses revealed that approximately 200 kinases have wide-open cysteine available at the active sites, which could use in covalent inhibition [57]. Kinase inhibitors are created by targeting various kinases, as shown in Fig. 2.

AKT is an AGC kinase and a type of protein kinase B that has been meticulously studied among protein kinases; recent studies show a comprehensive focus on developing small molecule kinase inhibitors based on AKT. It regulates cell proliferation, endurance, and apoptosis through downstream substrates like p21, p27, FoxO1, CREB, MDM2, GSK-3, procaspase-9, and Bad. Overactive AKT signaling is effective in bypassing apoptosis, allowing dysregulated cells to proliferate more freely, and making these cells resistant to radiotherapy in conjunction with chemotherapy [41]. Besides, AKT owns a PH domain (Pleckstrin Homology), which controls functions due to binding with a phosphatidylinositol lipid that enrolls AKT in the plasma membrane. External growth factors trigger AKT signaling, which stimulates the production of PIKs (phosphoinositide -3-kinases). Phosphatidylinositol 4,5-bisphosphate (PIP2), the second messenger, is phosphorylated by PI3K and converted to PIP3 that further connects to AKT at PH domain through electrostatic forces with the anionic phosphate groups present in PIP3 [58,59]. The allosteric AKT inhibitor MK-2206, a clinical phase II trial, is the product of further medication development. In patients with erlotinib resistance, a combination of MK-2206 with erlotinib for NSCLC (non-small cell lung cancer) demonstrated good results. Unlike prior ATP-competitive AKT inhibitors, this inhibitor family showed eminent selectivity against additional AGC family kinases, for instance, PDK1, PKA, PKC, SGK, and S6K. Surprisingly, these simply inhibited the comprehensive kinase and escaped the inhibition of the AKT kinase domain. Allosteric inhibitors like MK2206 are interposed between PH domains and kinases by establishing interactions like hydrogen bonding with both domains, as per the consequences of SAR analysis, protein X-ray crystallography, and mutagenesis experiments. These interactions keep the AKT in a tight conformation where the ATP binding site is both unaffordable and disarranged, blocking ATP and substrates to bind [60,61]. Another way to reduce AKT activity is to prevent PIP3 from activating it. Sulfonamides [62], API-1 [5], DPIEL [63], perifosine [64], and PIA23 [65] have been demonstrated to bind precisely with exalted affinity to AKT at the PH domain, simulating PIP3 with no change in AKT conformation from locked to unlocked. However, all such inhibitors have limited solubility, a high propensity for accumulation, and poor pharmacokinetics. Bisarylcyclohexanone, i.e., SC66, is another allosteric type AKT inhibitor that has been observed to block the activation and cell membrane translocation of AKT through PIP3 [66]. However, AKT is groomed with SC66 for ubiquitin-dependent post-translational reformation in the pericentrosomal region, followed by proteasomal destruction. Hence, demonstrating that ubiquitination of Lys63 might not help activate AKT in several instances of colon and breast cancer, the binding course of SC66 is unclear at present [18,67].

### ABL

The Tyr specific Abelson murine leukemia (Abl) viral oncogene analogue is another well-studied protein kinase named p150, JTK, c-Abl, Abl1. Dysregulation of Abl has been linked to apoptosis in cancer. ABL owns 1 cap with an N-terminal, 2 Src (SH2/3) homology domains, an actin-binding domain, and a DNA binding domain in addition to the kinase domain. Myristoylation of the N-terminal cap controls the autoinhibition of Abl by phosphorylation. GNF-2 binding caused Abl autoinhibition due to bending vibration of the α-I helix and the resulting configurationally stabilization of the kinase active site via the Sh2 and Sh3 domains [68–70]. Furthermore, GNF-2 enhances the effectiveness of the ATP-competitive antagonists, implying the possible contact of ATP pocket with the myristate. A larger and heavier ligand in the myristate groove, on the other hand, was found to provide an impact on the potential of Abl [68].

### AKT

Phosphoinositide-dependent kinase 1, an AGC lineage constituent, is a leading kinase and a stimulator for several AGC kinases involved in insulin and growth factor signaling, including SGK, AKT, PKC, and S6K. Unlike other kinases, PDK1 lacks a C-terminal hydrophobic motif for intramolecular binding to the hydrophobic channel. The hydrophobic pocket of PDK1 performs like an identification spot in favor of the hydrophobic motif of its substrate kinases [71]. Balendran et al. revealed that the hydrophobic groove of PDK1 exhibited interaction with the C-terminal present in PKC-allied protein kinase 2 (PRK2) and named as PDK1-interacting fragment (PIK). Consequently, the hydrophobic groove was quickly labeled as a PIF binding pocket [72].

### JNK1

The JNKs are c-Jun N-terminal kinases comprising three isoforms, JNK 1/2/3, which are the subdivision of MAP kinases termed “anxiety/stress-triggered kinase”. Accordingly, these have been linked to checking inflammatory responses by triggering cytokine expressions like eotaxin, GM-CSF, and IL-1 employing downstream targets such as p53, AP-1, ATF2, and BAD. Like a few other MAP kinases, JNKs are regulated by phosphorylation of the A-loop through higher kinases like MKK4/ MKK7. Stebbins et al. examined 30000 compounds using DELFIA, i.e. Dissociation Enhanced Lanthanide Fluoro-Immuno Assay, and determined a series of tiny compounds that interfere with JNK1-pepJIP1 binding [73]. Whereas several of these drugs compete successfully via pepJIP1 binding, and BI-78D3 like compounds were capable of subsiding JNK1 phosphorylation pursuit with IC50 value, both in the micromolar and nanomolar range [74].

### CHK1

CHK1 is a Ser/Thr type kinase known as checkpoint kinase 1 and is a key transducer that plays a role in the cell division cycle through the control of the DNA impairment response during mitosis. Academics, as well as the pharmaceutical sector, have created many conventional ATP-competitive CHK1 antagonists, but they have limited cellular efficacy. Following drug development and co-crystal structure, studies with CHK1 verified the inhibitors’ allosteric binding location, which included H-bonds alongside hydrophobic contacts to the exterior of the protein next to the αD-helix extant on the C-lobe. Unlike additional kinases, αD-helix of CHK1 comprises a PDIG array having N-terminal Pro that prompts a tight turn, causing the development of a superficial, vastly surface- uncovered groove that these inhibitors can target [75,76].

### IGF-1R

IGF-1R is an insulin growth factor receptor having two α-subunits (extracellular ligand-binding domains) and two β- subunits (cytoplasmic domain). Kinase and transmembrane domains make up the β-subunits. The tetramer of IGF-1R goes through conformational alterations in response to ligand binding, which causes auto-phosphorylation of its kinase activation loop, triggering an increase in the activity of IGF-1R for phosphorylation and MAPK activation along with signaling of PI-3K [77,78]. Sequences of indole-butyl amines having antagonistic activity towards IGF-1R at concentrations ranging from low to sub-micromolar have been developed by Heinrich et al. using high-throughput screening operations. MSC1609119A-1 also expands into the superficial groove at C-lobe, where it forms contacts with the activation loop, αC-helix, and various C-lobe remainders through a wide-ranging network of H-bonds facilitated by water. Such a novel binding approach opens the door to the growth of imminent allosteric inhibitors [79].

### CDK2

Cyclin-dependent kinases (CDKs) are specifically Ser/Thr type kinases, and CDK2 is the fundamental unit in the transition phase of the cell cycle from G1 to S-phase. Misregulation of CDKs can result in various diseases, like inflammation and cancer. Although, some kinases may adopt unique A-loop configurations like DFG-in/ out that are usually markers of their activity level, others, like CDK2, exhibit difficulty in adopting the DFG-out conformation [80,81]. Betzi et al. discovered ANS, i.e., 8-anilino-1-naphthalene sulfonate as a unique allosteric CDK2 antagonist in addition to a distinct mechanism of binding. The antagonistic efficacy of ANS was comparable to that of CDK2 activator cyclin A but not to that of ATP or Type I CDK2 inhibitors like JWS648. ANS in bimolecular form bind near to DFG motif present in a void produced through C-helix and the filaments of N-lobe like β3, β4, β5, which is still in DFG-in conformation. Major reconstitutions of the ATP binding pocket were needed to fit the two inhibitor molecules [82].

### mTOR

Phosphatidylinositol-3-kinase-related kinases, also known as Ser/Thr kinase mammalian target of rapamycin (mTOR) are a module of the PI3K/AKT/mTOR signaling cascade. The mTOR complex 1, comprising mLST8, PRAS40, & raptor; mTOR complex 2, comprising mSIN1, mLST8, & rictor, all possessing separate biologic functions, can build 2 separate complexes using various protein combinations [83]. Rapamycin, a natural substrate derived from *Streptomyces hygroscopicus*, acts as an allosteric inhibitor of mTORC1. It binds to the C-terminus of mTOR, also termed the FKBP12-rapamycin binding domain, via hydrophobic interactions with the 12-kDa FK506- binding protein, culminating in a complex that can limit mTORC1 function [84]. Due to rapamycin’s limited solubility and bioavailability, a variety of derivatives, also termed rapalogs, have been designed and investigated in clinical trials [85].

### The delivery system for kinase inhibitors

The major goal of nanomedicine is to ensure that the drug reaches the target site at an acceptable strength with little loss in activity or volume in the bloodstream. It can also lower the risk of cellular toxicity to neighboring tissues and prevent drug resistance. Extensive metallic nanomaterials, polymeric nanomaterials, and liposomal formulations can be used, with advantages and disadvantages based on costs, loading capacity, drug release, bioavailability, and stability. The biological effect and dispersion of nanoparticles can be influenced by their size, shape, constitution, and surface charge. NPs with sizes greater than 150 nm are typically built up in the liver, spleen, and lungs; however, these are not bioaccumulated and cleared by the kidneys. Small NPs with sizes less than 5 mm exhibit the opposite effect. The NPs of the middle range are less likely to become stuck in the lungs than those in the spleen and the liver. Spherical NPs are generally trapped in the liver, while rod-shaped NPs reside in the spleen and liver [86,87]. The cellular internalization of NPs is uncomplicated with small cellular NP interaction points, hence, oval, as well as spherical NPs, easily internalize as compared to lengthened NPs. The positive surface charge of NPS facilitates their filtration outside the bloodstream through the spleen, liver, and lungs, making them unsuitable for long-term blood circulation. However, the characteristics of NPs can be developed during circulation, and active targeting with improved absorption, using approaches such as a change in surface charge, addition of functional groups, and coating with addition [88–91].

Because of its great biodegradability and minimal immunogenicity, serum albumin has recently been hailed as an ideal nanocarrier. The proteinic atmosphere comprising the binding/adsorbent proteins surrounding the NPs (nanoparticles), was thought to exhibit a crucial impact on phagocytosis and retention throughout the blood circulation. As an endogenous component, Albumin can protect small molecule TKIs from undesirable interactions and rapid *in vivo* elimination. The nanoformulations made using albumin possessed superior anticancer activity, reducing preliminary breast cancer and tumor cell movement. Albumin nanoparticles had a greater cytotoxic effect on cancer cells and reduced the unfavorable effects of chemotherapeutic drugs in treatment, indicating better therapeutic results [92,93]. Petrushev et al. described the conjugation of medication-based tyrosine kinase inhibitors onto spherical gold nanoparticles and their successful transmembrane delivery inside acute myeloid leukemia cells. These were also evaluated for their therapeutic efficiency against cell lines like OCI-AML3 and THP1. They proposed using rituximab in gold nanoparticles for managing severe myeloid leukemia [94]. Acurin nanoparticles, developed for the kinase inhibitor AZD2811, and observed accumulation along with retention of the drug in tumors with no influence on bone marrow pathology, which led to decreased toxicity and enhanced efficacy at half dose strength AZD1152, a hydrophilic prodrug of AZD2811 [95]. Kinase target inhibitors in liposomal formulations possess the benefits of elevated drug loading through surface ligand reformation that will provide optimal therapeutic effectiveness in target therapy for NSCL cancer (non-small cell lung cancer) [96]. PEGylation of carriers reduced the serum protein adsorption on the nanoparticle surface, leading to an increase in circulation time and anticancer efficiency. Imatinib in PLGA nanoparticles with NRP-1 targeting inhibited Treg cells in the tumor microenvironment, resulting in effective tumor immunotherapy [97]. Correia et al. created nanocomposites with undecylenic acid using thermally hydrocarbonized permeable silicon NPs rich in the anticancer agent sorafenib. The surface was conjoined with heptakis (6-amino-6-deoxy)-β-cyclodextrin to illustrate the effect of exterior polymeric functionalization on the physical and biologic characteristics of NPs. These were found to exhibit excellent cellular proliferation in breast cancer [98].

## Conclusion

Kinases are a wide family of enzymes with various target locations. Regardless of a significant asset in this targeting area, only a portion of it has been realized. The target spaces as well as limited opportunities have been used to their full potential. Overall, while kinase inhibitors, like any other type of cancer therapy, have inherent flaws, and clinical trials with currently investigated kinase inhibitors are still a long way off, there are enormous promise and opportunity in TKI therapy for cancer and other disease treatments. Cell-specific administration of kinase inhibitors has not been attempted as extensively as that of other medications, possibly because kinase inhibitors were first offered as targeted therapies because they were considered to inhibit only one specific target.
